# The MEK2-binding tumor suppressor hDlg is recruited by E-cadherin to the midbody ring

**DOI:** 10.1186/1471-2121-12-55

**Published:** 2011-12-20

**Authors:** Suzanne Gaudet, Marie-Josée Langlois, Robert A Lue, Nathalie Rivard, Alain Viel

**Affiliations:** 1 Department of Cancer Biology and Center for Cancer Systems Biology, Dana-Farber Cancer Institute, 450 Brookline Avenue, Boston, MA 02215, USA; 2 Department of Genetics, Harvard Medical School, Boston, MA 02115, USA; 3 Department of Molecular and Cellular Biology, Harvard University, 16 Divinity Avenue, Cambridge, MA 02138, USA; 4 Department of Anatomy and Cell Biology, Faculté de Médecine et des Sciences de la Santé, Université de Sherbrooke, 3001 12th North avenue, Sherbrooke, Canada

## Abstract

**Background:**

The human homologue of the *Drosophila *Discs-large tumor suppressor protein, hDlg, is a multi-domain cytoplasmic protein that localizes to the membrane at intercellular junction sites. At both synaptic junctions and epithelia cell-cell junctions, hDlg is known to recruit several signaling proteins into macromolecular complexes. hDlg is also found at the midbody, a small microtubule-rich structure bridging the two daughter cells during cytokinesis, but its function at this site is not clear.

**Results:**

Here we describe the interaction of hDlg with the activated form of MEK2 of the canonical RAF/MEK/ERK pathway, a protein that is found at the midbody during cytokinesis. We show that both proteins localize to a sub-structure of the midbody, the midbody ring, and that the interaction between the PDZ domains of hDlg and the C-terminal portion of MEK2 is dependent on the phosphorylation of MEK2. Finally, we found that E-cadherin also localizes to the midbody and that its expression is required for the isoform-specific recruitment of hDlg, but not activated MEK2, to that structure.

**Conclusion:**

Our results suggest that like at other cell-cell junction sites, hDlg is part of a macromolecular complex of structural and signaling proteins at the midbody.

## Background

hDlg, the human homologue of the *Drosophila *Dlg tumor suppressor, is an alternatively spliced protein that belongs to the membrane-associated guanylate kinase (MAGUK) protein family. MAGUKs are characterized by several protein interaction domains: three PDZ domains, an SH3 domain, a guanylate kinase-like domain (GK), and a L27 self-association domain [1,2]. Most PDZ domains bind to the C-terminal portion of proteins often characterized by one of three consensus sequence classes: -X-(S/T)-X-Φ (Class I), -X-ΦX-Φ (Class II), -X-(D/E/K//R)-X-Φ (Class III) (where Φ represents an aliphatic residue; [3]), with all four terminal residues additively contributing to interaction specificity [4]. The three PDZ repeats of hDlg use this mechanism to bind to several proteins involved in cellular growth control including the adenomatous polyposis coli (APC) tumor suppressor [5,6], the human papillomavirus E6 protein [7], the adenovirus E4 protein [8], the mitotic Ser/Thr kinase PBK/TOPK [9], and p38γ MAP kinase [10]. The GK domain of hDlg also recruits several proteins into macromolecular complexes: GKAP/SAPAP [11,12], the PKA-targeting protein AKAP79/150 [13], and the microtubule-associated protein MAP1A [14]. The SH3 domain of hDlg forms an *intramolecular *interaction with the GK domain [15]. Finally, homo- and hetero-oligomers of MAGUK proteins form through their L27 domains; for example, hDlg and the MAGUK protein CASK heterodimerize through their L27 domains [16,17]. The degree of hDlg self-association depends also on the presence or absence of the alternatively spliced insertion I1A [18]. I1A and B, two proline-rich alternatively spliced insertions upstream of the first PDZ repeat in hDlg, recruit SH3-containing proteins [18].

First described as a cytoplasmic protein localized at the membrane at regions of intercellular contacts [2,19], hDlg is responsible for the recruitment of a variety of proteins forming a complex network at sites of epithelial cell-cell contact and in pre-synaptic densities. For example, hDlg has been found to be closely associated with E-cadherin in human intestinal epithelial cells ([20,21]. More recently, I2-containing alternatively spliced variants of hDlg have been reported to be found in the nucleus of cultured human cancer cells [18,22] and of cells from human epithelial tissues (AV, unpublished results), and both I3- and I2- containing variants were reported to localize to the midbody of cells in cytokinesis [23,24].

While the various localization sites of hDlg are known, it is unclear what its function is at those sites. An important step in understanding the function of hDlg as a tumor suppressor is the identification of all of its binding partners. Here we describe the interaction of hDlg with the phosphorylated form of MEK2, a signaling protein found, like hDlg, at the midbody of cells undergoing cytokinesis. Importantly, our data also indicate that E-cadherin concentrates in the midbody during cytokinesis and is necessary for proper localization of hDlg, but not phosphorylated MEK2, at the midbody.

## Results

### A C-terminal fragment of MEK2 interacts with hDlg

Like other members of the MAGUK family, hDlg plays an important role in clustering signaling molecules at sites of cell-cell contact. Most of the structural modules found in hDlg are known to function as protein-interaction domains. In an effort to identify new signaling proteins that bind to hDlg, we performed a two-hybrid screen using full-length hDlg as bait. This screen yielded many positives. Among the clones that most strongly activated the lacZ reporter gene was a cell cycle-regulated kinase [9] and a ~900 bp sequence encoding the C-terminal 126 residues of MEK2 (pGAD-MEK2(275-400)). Once isolated, this MEK2 construct was retransformed in *S. cerevisiae *HF7c with pGBT9-hDlg to confirm that the interaction was not due to another co-transforming plasmid (Table 1). The MEK2 construct was also co-transformed with a series of control plasmids to confirm that reporter gene activation was indeed dependent on a specific interaction with hDlg (Table 1); with this set of transformations we observed the same pattern of β-galactosidase activity that was found with a C-terminal clone of PBK, a previously identified hDlg-interacting protein [9].

**Table 1:** β-galactosidase activity of *S. cerevisiae *HF7c after co-transformation with indicated plasmid pair

Prey plasmid	Bait plasmid	Bait description	β-galactosidase activity	Data Source
pGAD-MEK2(275-400)	pGBT9-hDlg	full-length hDlg	+++	This paper
pGAD-MEK2(275-400)	pGBT9	vector only	-	This paper
pGAD-MEK2(275-400)	pLAM5'	human lamin C (66-320)	-	This paper
pGAD-MEK2(275-400)	none		-	This paper
pGAD-PBK(173-322)	pGBT9-hDlg	full-length hDlg	+++	[9]
pGAD-PBK(173-322)	pGBT9	vector only	-	[9]
pGAD-PBK(173-322)	pLAM5'	human lamin C (66-320)	-	[9]
pGAD-PBK(173-322)	none		-	[9]

**Figure 1:**
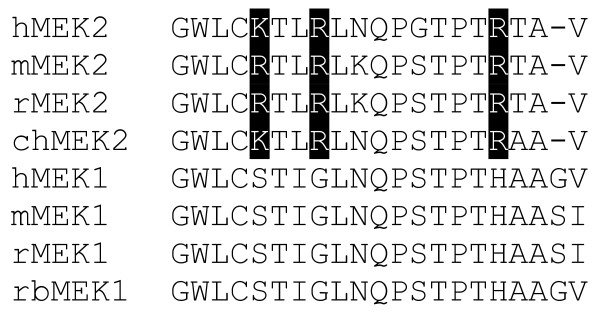
**Alignment of the C-terminal sequences of MEK1 and MEK2**. The last three residues of MEK2 proteins (with the exception of MEK2 from chicken) suggest that MEK2 is characterized by a Class I PDZ-binding motif. This motif is absent in MEK1 proteins. A set of three conserved basic residues (highlighted in black) is also a feature specific of MEK2 proteins. The sources for the aligned sequences are: human MEK2 (hMEK2, NP_109587), mouse MEK2 (mMEK2, AAH14830), rat MEK2 (rMEK2, 2113192A), chicken MEK2 (chMEK2, NP_990719), human MEK1 (hMEK1, NP_002746), mouse MEK1 (mMEK1, NP_032953), rat MEK1 (rMEK1, NP_113831), and rabbit MEK1 (rbMEK1, P29678).

Although only MEK2 was isolated in our screen, MEK1 and MEK2 share 80% sequence similarity [25]. Notably, their C-terminal sequences differ: only MEK2 is characterized by a conserved X-(S/T)-X-Φ motif matching the consensus sequence found at the C-terminus of Class I PDZ-binding proteins (Figure 1). Human, mouse and rat MEK2 all contain this motif, while in chicken MEK2, the threonine residue in position P-2 is replaced by an alanine (Figure 1). MEK1 sequences from all four species are characterized by this same Thr to Ala substitution and by the addition of glycine or serine at the penultimate position (Figure 1). The presence of a PDZ-binding motif in mammalian MEK2 proteins suggests that human MEK2 interacts with hDlg through one of its three PDZ repeats.

**Figure 2:**
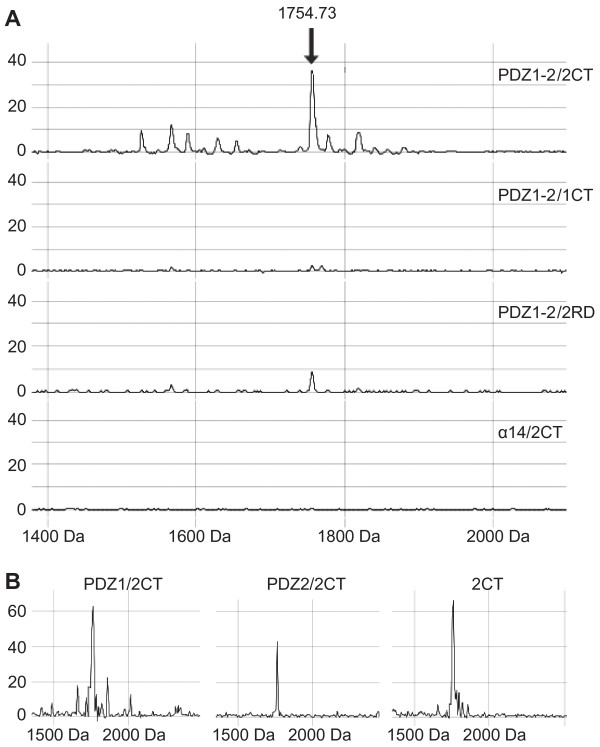
**The C-terminal sequence of MEK2 binds to the PDZ repeats of hDlg**. Peptides corresponding to the C-terminal end of human MEK2 (2CT) or human MEK1 (1CT), and a randomized version of 2CT (2RD) were assayed for binding to a polypeptide comprising PDZ1-PDZ2 of hDlg fused to GST (PDZ1-2). Bound fractions were analyzed by SELDI on H4 spots on a ProteinChip array (see material and methods). The area under the peak (arbitrary units) was proportional to the amount of bound peptide. (**A**) While 2CT bound to GST-PDZ1-2, no detectable peptide peak was observed when either 1CT or 2RD were tested for binding to GST-PDZ1-2; an assay with an unrelated GST fusion protein (α14) established the binding specificity of 2CT. (**B**) 2CT also bound to individual PDZ repeats fused with GST (PDZ1, left and PDZ2, middle). In this assay, the analysis of 2CT directly spotted on the ProteinChip array showed that protein-bound 2CT and free 2CT occupied the same position on MALDI spectra (right). Preliminary experiments showed that both 1CT and 2RD can be detected by SELDI (data not shown).

To test our hypothesis that the C-terminus of MEK2 but not that of MEK1 interacts with the hDlg PDZ repeats, we designed a peptide binding assay. In this assay, peptides corresponding to the last 16 residues of MEK2 (2CT) or MEK1 (1CT), or a randomized sequence of the MEK2 peptide (2RD) were incubated with a GST fusion protein containing PDZ1-PDZ2 of hDlg (GST-PDZ1-2), a fragment of hDlg that was previously identified as a super-motif [19]. MALDI-TOF analyses consistently showed that only the MEK2 peptide bound to GST-PDZ1-2 (Figure 2A). The peptide recovered after elution had a mass identical to the MEK2 peptide directly spotted on the ProteinChip array (Figure 2B, right). When the same experiment was repeated with an unrelated GST fusion protein (containing the 14^th ^repeat of α-spectrin, GST-α14), the MEK2 peptide did not bind, demonstrating the specificity of this peptide for PDZ1-2 of hDlg (Figure 2A). Binding of the MEK2 peptide was also observed when each of the three PDZ repeats of hDlg was expressed individually and used in our binding assay, although our results suggest that the binding is weakest with PDZ3 (Figure 2B and data not shown). This last result corresponds well with those of Maiga et al. (2011) who have also recently described an interaction of hDlg with MEK2 and found, in a two-hybrid assay likely less sensitive than our peptide binding assay, that a C-terminal construct of MEK2 (179-400) interacts with a PDZ1-2 construct but not with PDZ3. Overall, our peptide assay data confirmed that MEK2, but not MEK1, has a Class I PDZ-binding motif that interacts with the PDZ repeats of hDlg.

### hDlg interacts only with activated full-length MEK2

**Figure 3:**
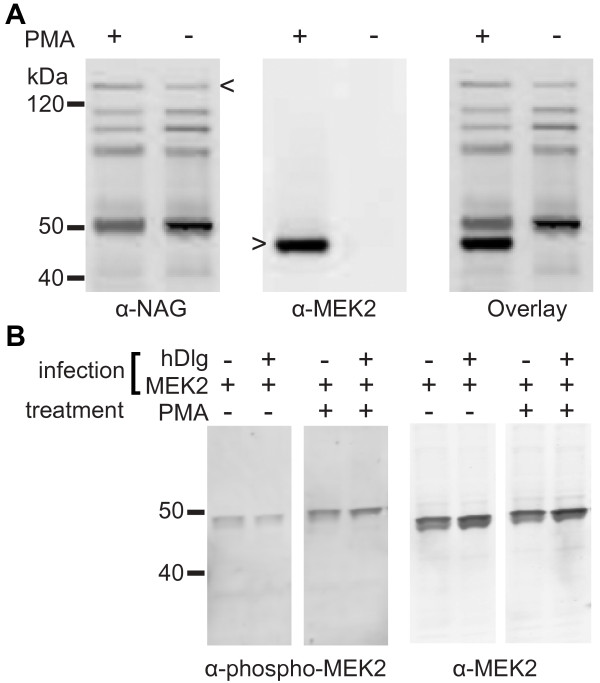
**hDlg interacts with activated MEK2**. A GST-hDlg fusion protein and MEK2 were co-expressed in insect cells. Lysates from PMA-treated (+) and untreated (-) cells were incubated with GSH-agarose and the content of the bound fractions was submitted to SDS-PAGE and transferred onto nitrocellulose. The membrane was probed with anti-hDlg (α-NAG) and anti-MEK2 (α-MEK2) antibodies. The membrane was scanned with a dual laser Odyssey system to co-detect hDlg (left panel, **A**) and MEK2 (middle panel, **A**). The right panel in A shows the overlay of the two scans. Full-length hDlg and full-length MEK2 are identified by arrow heads. The multiple bands detected by anti-hDlg antibodies correspond to breakdown products consistently observed when hDlg is overexpressed in insect cells. Panel **B **shows immunoblots of lysates from PMA-treated (+) and untreated (-) cells that were infected with baculovirus for the expression of MEK2 alone or in combination with baculovirus driving the expression of hDlg and probed with anti-MEK2 (α-MEK2, left) and anti-phospho-MEK1/2 (α-phospho-MEK2, right).

To confirm that hDlg and MEK2 interact *in vivo*, full-length hMEK2 was co-expressed with a GST-hDlg fusion protein in insect cells. Although co-infected insect cells synthesized both proteins, our initial attempts to show a co-purification of MEK2 and GST-hDlg failed. The co-localization of hDlg with the phosphorylated form of MEK2 during cytokinesis (see below) prompted us to test for the interaction upon stimulation of Raf/MEK/MAP kinase pathway. Abdullah et al. (1995, [26]) demonstrated that PMA-treatment of Sf9 insect cells activates this signaling pathway. We co-infected High 5 insect cells with recombinant baculoviruses responsible for the expression of GST-hDlg and hMEK2. GST-hDlg was affinity purified from untreated and PMA-treated insect cell lysates and MEK2 co-purification was assayed by immunoblot. While the amounts of GST-hDlg affinity-purified in untreated and PMA-treated samples were comparable, hMEK2 co-purified with hDlg only in PMA-treated samples (Figure 3A). Both treated and untreated lysates expressed hMEK2 at similar levels although hMEK2 phosphorylation level was significantly increased by PMA treatment (Figure 3B). Our data shows that MEK2 is not pulled down non-specifically by glutathione beads and suggest that in this cellular context hDlg interacts very selectively with the activated form of MEK2, which is present only in PMA-treated cells. Nevertheless, we cannot rule out that the activation of the Raf/MEK/ERK pathway resulted in the phosphorylation of other proteins, including hDlg, and that this may also contribute to the observed interaction.

### hDlg localizes to the midbody ring during cytokinesis

**Figure 4:**
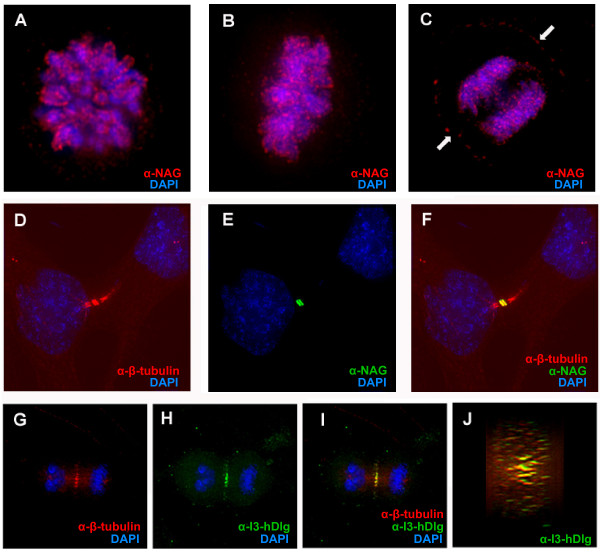
**Distribution of hDlg during mitosis**. MCF10A cells at different stages of mitosis were stained with antibodies directed against total hDlg (all variants, α-NAG, Panels **A**-**F**) or antibodies raised against the alternatively spliced insert I3 (Panels **G**-**J**); both antibodies have previously been extensively validated for specificity against hDlg [18,44]. Cells were co-stained with antibodies directed against β-tubulin (Panels **D **and **G**). The image overlays in panels **F**, **I**, and **J **show the relative distribution of hDlg (green) and microtubules (red). These images were produced by deconvolution of contiguous Z-sections. Panel J shows a reconstruction of a cross-section of the midbody ring structure co-stained with anti-I3 (green) and anti-β tubulin (red) antibodies. This cross-section is orthogonal to the central spindle. DNA (blue) was visualized by DAPI staining. The white arrows (Panel **C**) indicate the position of the cell membrane. All scale bars are 10 μm.

Because activated MEK2 is reported to localize to spindle poles [27-29], kinetochores [29,30] and to the central portion of the midbody in mitotic cells [31], we next examined the localization of hDlg during various stages of mitosis. MCF10A human breast carcinoma cells were stained with affinity purified anti-hDlg (anti-NAG) and anti-β-tubulin antibodies. The mitotic cells were staged based on the appearance of their spindle in the β-tubulin staining and that of their chromosomes stained by DAPI, as well as from phase-contrast imaging of the cellular membrane. From prophase to anaphase, hDlg was distributed at the surface of condensed chromosomes (Figure 4A-C) and at the cell periphery (Figure 4C, arrows). I2-containing hDlg variants were previously observed in the interphase nuclei of a variety of cells types including MCF10A cells ([18] and AV, unpublished data). During mitosis, it appears that the nuclear variants of hDlg were not dispersed in the cytoplasm but, instead, remained associated with condensed chromosomes, distributed in discrete spots along their entire length (Figure 4A-C). A set of contiguous Z-sections was used to create a three-dimensional rendering of the cell shown in Figure 4C. A 360° rotation of this rendering showed that there was no polarity in the distribution of hDlg at the surface of metaphase chromosomes; it was also present at the sides of the two sets of anaphase chromosome facing each other (Figure 4C). Based on this staining distribution, we conclude that hDlg was not specifically associated with telomeric or kinetochore proteins in mitotic cells, but spread all over the chromosomes.

In cells in cytokinesis, midbody structures were easily detected by immunostaining with anti-β-tubulin (Figure 4D). Co-staining with anti-NAG showed that hDlg was located in the central portion of the midbody called the midbody ring, and was not distributed along the bundles of microtubules linking the two nascent daughter cells (Figure 4E). Our data confirms similar observations of hDlg at the midbody in HaCat skin keratinocytes, Saos2 and U2OS osteosarcoma cell lines by Massimi et al. (2003; [23]) and in U2OS and HeLa cervical carcinoma cells by Unno et al. (2008, [24]) although we found that the localization of hDlg is restricted to the midbody ring. A few proteins, such as β-tubulin, centriolin and activated MEK2, are known to associate specifically with the midbody ring while others, such as Aurora B, are found adjacent to the ring structure ([31,32] and our unpublished data). This co-localization of activated MEK2 and hDlg to the very limited structure of the midbody ring suggests that hDlg could recruit or anchor activated MEK2 in a particular signaling complex at the midbody.

To verify that the distribution of hDlg that we observed did not simply reflect an unregulated or non-specific interaction with tubulin subunits or microtubule-associated proteins, we looked more closely at the co-staining of midbody structures with anti-hDlg and anti-β-tubulin antibodies in cells at late stages of cell division. The distribution of hDlg during late anaphase or early cytokinesis showed that it localizes near microtubules plus ends (Figure 4G-J). Before constriction of the cytoplasmic bridge, hDlg was distributed in a narrow line enriched in tubulin in the middle of the central spindle (Figure 4G-I). A cross-section of the midbody ring structure reconstituted from contiguous Z-sections showed that, before membrane constriction, hDlg was concentrated at the center of the ring structure rather than distributed at its periphery (Figure 4J). The overlap between tubulin and hDlg staining was only partial and in most cases hDlg appeared juxtaposed to microtubules (Figure 4J), clearly showing that the association of hDlg with the midbody ring is not simply due to an unregulated association with tubulin.

Finally, when staining with an antibody that specifically recognizes the membrane-associated hDlg variants (containing the I3 alternatively spliced insert [18]) we found that they localized to the midbody ring (Figure 4G-J). Strikingly, staining with affinity purified antibodies raised against the alternatively spliced insertion I2, present in nuclear variants of hDlg, failed to label this structure (data not shown). Therefore we conclude that only the membrane-bound form of hDlg was recruited to the midbody, where it is found specifically at the midbody ring, near microtubule plus ends. In contrast, the I2-containing variants remains associated with the chromosomes. This isoform-specific localization of endogenously expressed hDlg contrasts with the non-specific localization of both I3- and I2-containing isoforms others have found for overexpressed full-length or truncated C-terminal forms of hDlg [23,24].

### E-cadherin is necessary for localization of hDlg but not phosphorylated MEK2 to the midbody ring during cytokinesis

**Figure 5:**
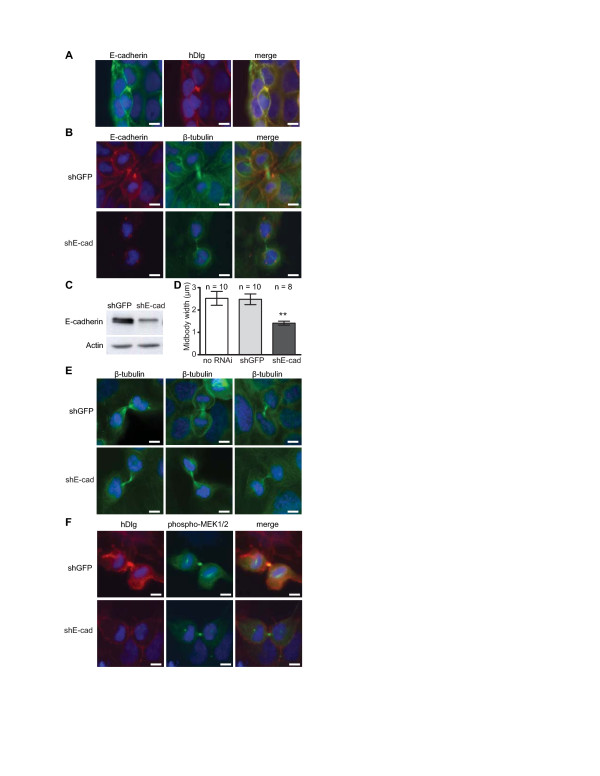
**Impact of E-cadherin downregulation on localization of hDlg and activated MEK2 at the midbody**. Subconfluent Caco-2/15 cells were stained with antibodies directed against total hDlg (α-NAG, red) or against E-cadherin (green) (Panel **A**). Caco-2/15 cells stably infected with lentiviruses encoding for a control shRNA (shGFP) or encoding an E-cadherin-specific shRNA were lysed and proteins were analyzed by Western blotting for expression of E-cadherin and β-actin (Panel **C**). Caco-2/15 cells stably expressing shGFP or shE-cadherin were stained with antibodies directed against E-cadherin (red) and β-tubulin (green) (Panel **B**), against β-tubulin alone (green) (Panel **E**) or against hDlg (Santa Cruz antibody, red) and phospho-MEK (green) (Panel **F**). In all images, DNA (blue) was visualized by DAPI staining and scale bars are 10 μm. Midbody widths were determined at their central portion for cells that were not infected (no RNAi), or stably expressing lentiviral-coded control (shGFP) or E-cadherin specific shRNAs (Panel **D)**. Error bars represent the standard deviation across the population of cells (number of cells for each sample is indicated on the graph); asterisks indicate that the average for E-cadherin shRNA-expressing cells is statistically different from that of both control cell populations (two-tailed Student t-test) at p ≤ 0.01.

We have previously reported that hDlg is closely associated with E-cadherin adhesion complexes in epithelial cells [21]. Interestingly, staining for both E-cadherin and hDlg in subconfluent growing Caco-2/15 colorectal epithelial cells showed that both proteins co-localized at the midbody ring during cytokinesis (Figure 5A). To investigate whether E-cadherin controls the localization of hDlg and phosphorylated MEK2 to the midbody, E-cadherin expression was knocked down with RNA interference (Figure 5B-C). Reduction in E-cadherin levels markedly attenuated hDlg staining to the midbody (Figure 5B, F). With both E-cadherin and hDlg missing from the midbody, it appeared as if its structure was altered, as we consistently observed that the width of β-tubulin staining was reduced along the central spindle (Figure 5E). Although the midbody size in both control cells (no RNAi treatment and shGFP-transfected) and E-cadherin knockdown cells was variable, we found that on average, there was a clear, and highly statistically significant difference in the average midbody width (two-tailed Student't t-test, p = 0.002 for shE-cadherin vs. shGFP, p = 0.007 for shE-cadherin vs. no RNAi; Figure 5D). Somewhat surprisingly however, midbody staining for phosphorylated MEK2 was not affected by E-cadherin downregulation (Figure 5F). From these results we conclude that while E-cadherin is necessary for the localization of the I3-containing isoform of hDlg to the midbody ring during cytokinesis, the presence of neither of these proteins at the midbody structure is necessary for the recruitment of phosphorylated MEK2.

## Discussion

Although hDlg is known to localize to sites of cell-cell contacts, to interphase nuclei, and to the midbody of cells in cytokinesis [2,18,23], little is known about its binding partners at sites other than cell-cell contacts. Here we report that hDlg associates with activated MEK2, a protein specifically found at the midbody ring during cytokinesis [31]. Using *in vitro *binding assays, we found that the PDZ domains of hDlg interact directly with the C-terminal peptide of MEK2, which contains a Class I consensus PDZ-binding motif. Interestingly, while others have very recently shown partial co-precipitation of hDlg and MEK2 from asynchronous HEK-293 and human vascular endothelial cell lysates [33], in the cellular contexts we tested, the association between hDlg and full-length MEK2 is only detectable when MEK2 is activated. Several lines of evidence, including these data, suggest that the C-terminal PDZ-binding motif of full-length MEK2 may only be accessible to hDlg in the activated protein. Structural studies have shown that upon activation, MEK2 likely undergoes a change of conformation common to other kinases [34,35]. This change of conformation results in the relative rotation of the N- and C-terminal lobed-structures forming the catalytic domain. It is unknown how this rotation affects the C-terminal PDZ-binding sequence of MEK2 because the C-terminal seven amino acids, including this region, were not well ordered in the MEK2 structure [35]. Nevertheless, the crystallographic structure of MEK2 bound to Mg-ATP and a small molecule non-competitive inhibitor, PD334581, clearly shows that in this active-conformation homodimer, the PDZ-binding sequences should be accessible to the PDZ repeats of hDlg. Although not structured, the C-terminal seven residues would extend at the periphery of the complex, near the ATP binding site, and not be buried in the dimer interface [35]. Further supporting our hypothesis, we note that both the MEK2 clone isolated in our two-hybrid screen and the clone identified in a similar screen by Magai et al. (2011, [33]) coded for only the C-terminal portion of MEK2. Therefore in assays using a C-terminal fragment of MEK2 (Ref. [33] and herein), or a C-terminal MEK2 peptide (herein), the C-terminal PDZ-binding motif is likely more exposed than in full-length MEK2 clones, allowing for an interaction that is normally regulated by a phosphorylation-driven conformational change in the full-length protein. While our results and those of Maiga et al. [33] provide some initial information as to the binding specificities and the mode of interaction between hDlg and MEK2, a better understanding of how, and in which context, these two proteins can interact will require further structural studies. Other unresolved questions include how the intramolecular interaction between the GK and SH3 domains of hDlg, the self-association of hDlg proteins via their N-terminal ends, and the identity of hDlg splice variants contribute to the regulation of its binding to MEK2.

The role of MEK/ERK-dependent signaling in the control of cell-cycle has been extensively studied, particularly its role in G_1_/S phase transition. Recent studies have shown that activation of this signaling pathway is also required for mitotic progression. During late anaphase and cytokinesis, the two nascent daughter cells are separated by a cytoplasmic bridge characterized by a bundle of antiparallel microtubules forming the central spindle. At the half-point of the central spindle, a structure called the midbody determines the site of cell abscission and recruits proteins that are necessary for the completion of mitosis. The activated forms of MEK1/2, ERK and the ERK substrates RSK1-3 are found in the central spindle and in the midbody [29-31]. Inhibition of MEK activity prevents the completion of mitosis in cells released from nocodazole-mediated mitotic arrest, thus suggesting the role of MEK1/2 and downstream substrates in cytokinesis [31,36]. The interaction observed between hDlg and activated MEK2 suggests that both proteins should co-distribute at specific stages of the cell cycle when MEK2 is activated. Indeed, we have found that certain variants of hDlg, like activated MEK2, localize specifically to the midbody ring structure during cytokinesis.

It is unclear how hDlg is targeted to the midbody, however, because the PDZ domains of hDlg are insufficient to target the protein to the midbody [23], it cannot rely on activated MEK2. Others have shown that a protein fragment spanning only the C-terminal SH3 and GK domains of hDlg localized to the midbody when overexpressed, competing with endogenous hDlg and disrupting cell growth [23]. The GK domain of hDlg alone localizes to the midbody and causes a mild cytokinesis defect when overexpressed [24]. This suggests that while the N-terminal region of hDlg, including its PDZ domains, may be dispensable for proper localization of hDlg at the midbody, it is essential to its function.

A possibility raised by our data is that membrane-bound I3-variants of hDlg are redistributed to the midbody. I3-variants of hDlg were previously found to be localized to the membrane at sites of cell-cell junctions [19]. Membrane-bound hDlg was demonstrated to be closely associated with E-cadherin-containing adhesion complexes in human epithelial cells and cells deficient in hDlg expression fail to organize their cortical actin cytoskeleton and are unable to stabilize their adherens junctions [21]. E-cadherin protein was also previously found in the cleavage furrow of mitotic epithelial cells, consistent with the observation that the daughter cells form adherens junctions with each other immediately following cytokinesis and are not separated by neighboring cells [37]. Herein, we demonstrate that E-cadherin expression is required for localization of hDlg to the midbody in the same epithelial cells. E-cadherin depletion also led to a marked narrowing of the midbody structure. While our data does not specifically address whether E-cadherin depletion itself or its effect on the central spindle are the direct cause of the mis-localization of hDlg, because E-cadherin and hDlg are known to physically interact in confluent epithelial cells [21], we surmise that E-cadherin may directly recruit hDlg to the midbody. Taken together, these data suggest that hDlg may also be important for the formation of adherens junction between the two daughter cells.

Membrane-bound I3-containing variants of hDlg could either relocalize to the midbody from existing adherens junction sites or be recruited to the midbody from intracellular membrane systems. In this report, we show that the localization of hDlg to the midbody ring precedes the constriction of the intracellular cytoplasmic bridge, while post-Golgi secretory vesicles are delivered to the site of abscission after membrane constriction, during the late stage of cytokinesis [32], arguing against recruitment from intracellular membranes. Although only I3-variants of hDlg are preferentially associated with membrane systems [18,19], others have shown that the transport and/or anchoring of overexpressed hDlg to the midbody is independent of I2 and I3 [23,24]. Taken together our findings and those from these previous reports are consistent with the idea that SH3- or GK-binding proteins are responsible for the transport and or anchoring of hDlg to the midbody, while alternative splicing of the I3- vs. I2-insertion may determine which pool of hDlg relocalizes to this site, perhaps by regulating the accessibility of the GK domain.

hDlg plays an important role in clustering signaling molecules at different intracellular sites. In this report, we describe the interaction of hDlg with MEK2 which was also reported recently by Maiga et al. [33]. In contrast to hDlg, however, the recruitment of phosphorylated MEK2 to the midbody seems to occur normally in E-cadherin-depleted cells indicating that although other assays show that hDlg can bind directly to phosphorylated MEK2, hDlg is not required for MEK2 recruitment to the midbody. Nevertheless, we cannot exclude that hDlg may serve as a scaffold to form a signalosome that is responsive to MEK/ERK signaling during cytokinesis. Previous studies have identified several other kinases that bind to hDlg. First, PBK/TOPK, a kinase which is activated during mitosis by the cyclin B/CDK1 complex and then phosphorylates p38-MAPK, interacts with the PDZ repeats of hDlg [9,38]. Interestingly, an activated form of PBK/TOPK was recently found to be associated with the central spindle and to promote cytokinesis [39]; this association with the central spindle may reflect its interaction with hDlg. A second kinase interacting with hDlg is p85/PI3K, which it recruits to E-cadherin-mediated sites of cell-cell contact in human intestinal epithelial cells where hDlg plays a key role in the organization and stabilization of adherens junctions [21]. Importantly, this recruitment of p85/PI3K is dependent on a change in the phosphorylation pattern of hDlg triggered by a yet unidentified kinase [21]. Finally, the SAPK3/p38γ MAPK is also known to interact with the PDZ repeats of hDlg and to phosphorylate hDlg. Phosphorylation of hDlg by SAPK3/p38γ MAPK results in its dissociation from the cytoskeleton, apparently by disrupting the interaction between the GK domain of hDlg and its binding partner GKAP [10]. Therefore, not only is hDlg implicated in the recruitement of several kinases at specialized sites in cells but, in turn, the activity of hDlg, and potentially its localization, is modulated by kinases.

## Conclusions

In summary, we show that specific variants of hDlg associate with the midbody ring structure during late anaphase and cytokinesis and that the midbody localization of hDlg is dependent on the expression of E-cadherin. A clear understanding of the functions that hDlg performs at different intracellular sites requires the identification of all its interaction partners. We demonstrate that hDlg directly interacts with a component of the MAP kinase pathway, MEK2, specifically in cells where MEK2 is activated. Activated MEK2 is known to also localize to the midbody ring structure during late mitosis. Future studies will reveal how hDlg and MEK2 work together to regulate cell cycle events and cellular proliferation.

## Methods

### Two-hybrid screen

A two-hybrid screen was performed using the MatchMaker system (Invitrogen). A clone of *S. cerevisiae *HF7c transformed with the pGBT9-hDlg construct (HF7c/hDlg) was selected based on its expression of hDlg as detected by immunoblot. HF7c/hDlg cells were then transformed with 250 μg of the pGAD-GH HeLa cDNA library (Invitrogen) using lithium-acetate. The transformants were plated on SD (Leu^-^, Trp^- ^and His^-^) medium supplemented with 5 mM 3-amino triazole and incubated 7 days at 30°C. Of approximately 750,000 transformants, 2010 were HIS3^+ ^and of these, 236 were reproducibly positive for β-galactosidase activity. The transformants with the strongest β-galactosidase activity were further validated and tested for false positives by isolating plasmid DNA and transforming single clonal plasmid DNA in HF7c or co-transforming with pGBT9, pLAM5' and finally with pGBT9-hDlg.

### Cell culture and reagents

Sf9 and High 5 insect cells (Invitrogen) were grown in Grace's media supplemented with 10% fetal bovine serum and in BaculoGold protein-free insect cell medium respectively (Invitrogen). MCF10A human immortalized mammary epithelial cells (ATCC, Manassas, VA) were grown in Ham's DMEM/F12 supplemented with 0.1 μg/ml cholera toxin, 12 μg/ml insulin, 0.5 μg/ml hydrocortisone, 0.02 μg/ml epidermal growth factor, and 5% chelexed horse serum (Invitrogen) under a 5% CO_2 _atmosphere at 37°C. The Caco-2/15 human colon adenocarcinoma cells were obtained from Dr A. Quaroni (Cornell University, Ithaca, NY) and cultured in DMEM containing 10% FCS, as described previously [40].

### Protein co-expression in insect cells

A cDNA coding for a full-length variant of hDlg lacking alternatively spliced insertions I1A and I1B but containing insertions I3 and I5 [18] was subcloned into the baculovirus transfer vector pAcGHLT-B (Pharmingen). Recombinant baculoviruses were produced by the co-transfection of Sf9 cells with pAcGHLT-B/hDlg and BaculoGold DNA (Pharmingen).

Human MEK2 cloned into pCMV (kindly provided by Dr. Kun-Liang Guan, University of Michigan, Ann Arbor) was used as template to produced a PCR product coding for full-length MEK2. This PCR product was subcloned into the pBlueBak4.5/V5-His-TOPO baculovirus transfer vector (Invitrogen). Recombinant baculoviruses were produced by the co-transfection of Sf9 with pBlueBak/MEK2 and Bac-N-Blue triple cut DNA (Invitrogen).

High 5 insect cells were infected at high multiplicity of infection with hDlg and MEK2 recombinant baculovirus alone or in combination. Infected cells were harvested after three days and cell pellets were stored at -80°C until further processing. In some cases, infected High 5 cells were stimulated with 1 μM phorbol 12-myristate 13-acetate (PMA) for 1 h at 27°C before harvesting.

### Protein purification

cDNAs coding for hDlg PDZ repeats 1 and 2 or α-spectrin repeat 14 were cloned into a modified version of the expression vector pGEX (Pharmacia) and GST fusion proteins GST-PDZ1-2 and GST-α14 were expressed and purified as described previously [19,41].

To isolate GST-hDlg and its associated proteins, pellets of infected High 5 cells were resuspended in 1 to 1.5 ml of insect cell lysis buffer supplemented with a cocktail of protease inhibitor (Pharmingen) and phosphatase inhibitors (sodium orthovanadate and sodium fluoride). Samples were placed on ice for 30 min and then homogenized in a tissue grinder. The lysate was clarified by centrifugation at 14,000 rpm for 30 min, and then incubated with GSH-agarose beads (Sigma-Aldrich) for 1 h at 4°C. The GSH-beads were then washed three times with PBS supplemented with 0.01% Tween 20 and phosphatase inhibitors. The isolated proteins were eluted from the beads with SDS-PAGE sample buffer.

### Peptide binding assays

Peptides corresponding to the C-terminal portion of MEK2 (2CT: KTLRLNQPGTPTRTAV), MEK1 (1CT: TIGLNQPSTPTHAAGV), and a randomized version of the 2CT peptide sequence (2RD: RKTLGNRPPLVTTAQT) were synthesized by the peptide synthesis core facility (Massachusetts General Hospital CNY). Purity and quality of the synthesized peptides was confirmed by reverse-phase chromatography and MALDI-TOF.

40 μg of GST-PDZ1-2 or 90 μg of GST-α14 fusion proteins bound to 20 μl GSH-agarose beads were incubated with 8 μg of peptides for 2 h at 4°C. The beads were then washed twice with PBS-0.01% Triton X-100, and three times with 50 mM Tris, pH 7.6. Bound peptides were then eluted from the agarose beads by two sequential 5 min incubations, each with one bead-volume of 30% acetic acid.

To analyze the eluted peptides, spots on H4 ProteinChip arrays (Ciphergen) were pretreated with 50% acetonitrile, and then 4 μl of an eluted fraction was applied to each spot and allowed to dry. Next, 0.5 μl of a saturated solution of the energy absorbing α-cyano-4-hydroxycinnamic acid (CHCA, Sigma-Aldrich) diluted 1:5 in 50% acetonitrile and 0.5% trifluoroacetic acid was applied to each spot. Adsorbed peptides were detected using the ProteinChip Biology system reader IIC (Ciphergen).

### Immunoblots

To analyze the lysates of cells infected with baculoviruses coding for the expression of MEK2 (with or without hDlg co-expression, and with or without PMA treatment) as well as the proteins complexes co-purified with GST-hDlg from High 5 insect cells, the lysates or proteins affinity purified with GSH-agarose beads were resolved by reducing and denaturing electrophoresis on a 10-20% tricine gel, and then transferred onto nitrocellulose membranes. The membranes were blocked for 2 h at room temperature with Odyssey blocking buffer (Li-Cor Biosciences) and probed overnight at 4°C with mouse anti-MEK2 (Santa Cruz Biotechnology Inc) and rabbit anti-hDlg (anti-NAG, [18]) or rabbit anti-phospho-MEK1/2 (Cell Signaling Technology) primary antibodies. After washing, the membranes were incubated with Alexa 680-conjugated goat anti-rabbit (Molecular probes) and IRDye 800-conjugated goat anti-mouse (Rockland immunochemicals, Inc.) secondary antibodies. Membranes were analyzed with the Odyssey Imaging system (Li-Cor Biosciences).

To measure E-cadherin expression levels in Caco-2/15 cells, the cells were lysed in SDS-sample buffer (62.5 mM Tris-HCl [pH 6.8], 2.3% SDS, 10% glycerol, 5% β-mercaptoethanol, 0.005% bromophenol blue, 1 mM PMSF). Protein concentrations were measured using a modified Lowry procedure with BSA as standard [42]. Equal amounts of proteins from whole cell lysates were separated by SDS-PAGE in 10% gels and electrotransferred onto PVDF membranes (PerkinElmer). Membranes were blocked for 1 h at 25°C in PBS containing 5% powdered milk and 0.05% Tween-20 and then incubated overnight at 4°C with mouse anti-E-cadherin (BD Biosciences) or mouse anti-actin (Chemicon) primary antibodies followed by incubation with horseradish peroxidase-conjugated goat anti-mouse IgG (GE Healthcare) for 1 h at 25°C. The blots were visualized by the Amersham ECL system.

### Immunostainings

MCF10A cells grown on glass coverslips were washed with PBS and fixed at least 1 h in cold methanol. Fixed cells were rehydrated in PBS, and then incubated in primary antibodies in PBS with 3% BSA for 1 h at 37°C. After washing three times in PBS, the cells were incubated with the secondary antibodies in PBS with 3% BSA for 30 min at 37°C. The cells were washed once in PBS, stained 5 min at room temperature with 1 μg/ml DAPI in PBS and then washed twice in PBS before mounting in Prolong anti-bleaching agent (Invitrogen). We used affinity purified anti-NAG, affinity purified anti-I3 [18], and anti-β-tubulin (Santa Cruz Biotechnology Inc.) as primary antibodies and Cy5-conjugated goat anti-mouse (Zymed) or FITC-conjugated donkey anti-rabbit (Zymed) as secondary antibodies. Images were captured using an Applied Precision deconvolution microscope. The Z-series were deconvolved using SoftWoRx (Applied Precision Inc., Seattle, WA) and shown as maximum intensity quick projections.

Subconfluent Caco-2/15 cells grown on glass coverslips were washed twice with ice-cold PBS and fixed in paraformaldehyde (3%) for 20 min at room temperature. Fixed cells were then permeabilized with 0.1% Triton X-100 in PBS for 10 min and blocked with PBS-BSA 2% (30 min at 25°C). Cells were then incubated 2 h at 25°C with mouse anti-E-cadherin (Chemicon), rabbit or mouse anti-hDlg (α-NAG and mouse anti-Dlg1 from Santa Cruz Biotechnologies, respectively), goat anti-β-tubulin (Abcam) or rabbit anti-phospho-MEK (Cell Signaling Technology) followed by 30 min at 25°C with Alexa 488 and Alexa 568-conjugated secondary antibodies (Invitrogen). The cells were finally stained 5 min at 25°C with 1 μg/ml DAPI in PBS. Negative controls (no primary antibody) were included in all experiments. Images were captured using a Leica DM STC microscope using either a 20X or a 40X objective (Leica Instruments). Magnification was calibrated by comparison with a stage micrometer (Graticules™ Ltd., Tonbridge, UK). Midbody widths at their central portion were determined using the MetaMorph program (Universal Imaging Corporation, Sunnyvale, CA, USA). Statistics were calculated using the Student's two-tailed t test.

### RNA interference

The shRNA against E-cadherin (TRCN0000039666) and the control shRNA against TurboGFP^TM ^(SHC004) were obtained from Sigma-Aldrich. All lentiviruses were produced and used for cell infection according to Invitrogen recommendations (ViraPower Lentiviral Expression System, instructions manual). In each experiment with lentiviruses, *OAS1 *gene expression was analyzed by Q-PCR analysis. *OAS1 *(2'5'-oligoadenylate synthetase) is a classic interferon target gene and it has been recommended as a key test for interferon induction before attributing a particular response to the gene targeted [43]. No induction of *OAS1 *expression was detected in the experiments involving lentiviruses infection (data not shown).

## Authors' contributions

SG, MJL, RAL, NR and AV conceived and designed the experiments. SG, MJL and AV performed the experiments. SG, MJL, NR and AV analyzed the data. SG, MJL, RAL, NR and AV contributed to writing the paper and read and approved the final manuscript.
